# Auswirkungen der COVID-19-Pandemie auf die robotische Viszeralchirurgie in Deutschland

**DOI:** 10.1007/s00104-022-01684-x

**Published:** 2022-07-12

**Authors:** Jessica Stockheim, Mihailo Andric, Sara Acciuffi, Sara Al-Madhi, Mirhasan Rahimli, Maximilian Dölling, Gernot Geginat, Aristotelis Perrakis, Roland S. Croner

**Affiliations:** Klinik für Allgemein‑, Viszeral‑, Gefäß- und Transplantationschirurgie, Otto-von-Guericke Universität Magdeburg, Leipziger Str. 44, 39120 Magdeburg, Deutschland

**Keywords:** Robotik, Ausbildung, Onkologie, Kolorektal, HPB, Robotics, Training, Surgical oncology, Colorectal, HPB

## Abstract

**Einleitung:**

Der Einsatz roboterassistierter Operationen verzeichnet in der Viszeralchirurgie gegenwärtig einen stetigen Zuwachs. Im Jahr 2020 hat die COVID-19-Pandemie den klinischen und chirurgischen Alltag unerwartet wesentlich verändert. Wir haben in einer Umfrage den Status der roboterassistierten Viszeralchirurgie in Deutschland sowie die gegenwärtigen Ausbildungskonzepte evaluiert und deren Veränderungen unter dem Einfluss der COVID-19-Pandemie untersucht.

**Material und Methoden:**

In einer umfangreichen Recherche wurden 89 Kliniken identifiziert, welche ein Robotersystem für die Viszeralchirurgie 2020 einsetzten. Diese Kliniken wurden über eine webbasierte anonyme Umfrage mit 35 Fragen dreimal kontaktiert. Die Fragen bezogen sich auf die Einsatzgebiete eines Operationsroboters in der Viszeralchirurgie, die dazugehörige klinische Ausbildung und den Einfluss der COVID-19-Pandemie auf das bestehende Programm.

**Ergebnisse:**

Von den angeschriebenen Kliniken haben 22 (24,7%) eine Rückmeldung gegeben. Hiervon waren 17 (19,1%) Fragebögen auswertbar. Es beteiligten sich 58,8% Universitätsklinika, 17,6% Maximalversorger und 23,5% Schwerpunktkrankenhäuser an der Studie. Der Operationsroboter wurde am oberen Gastrointestinaltrakt (OGIT; 88,2%), am hepatopankreatikobiliären System (HPB; 82,4%) und im kolorektalen Bereich (KRK; 94,1%) sowie bei der Hernienversorgung (35,3%) eingesetzt. Der relative Anteil robotischer Eingriffe am operierten Gesamtkollektiv lag dabei zwischen 0,3% und 15,4%. Die Konversionsraten für 2020 lag im Mittel bei 4,6 ± 3,2%. Die Operationsroboter wurden zum Großteil im interdisziplinären Setting wechselweise mit anderen chirurgischen Disziplinen (82,4%) genutzt. Zu Lehrzwecken stand in sieben Kliniken (41,2%) eine zweite Konsole zur Verfügung. Die Ausbildungsstrukturen waren sehr heterogen und nur 13,2 ± 6,5% der Chirurg*innen pro Klinik waren in das Roboterprogramm involviert. In 82,4% existierten feste Teams, die sich aus Ober‑, Fach- und Assistenzärzt*innen zusammensetzen und in 76,5% wurden Ärzt*innen und Pflegepersonal über klinikinterne Ausbildungsprogramme geschult. Die COVID-19-Pandemie hatte einen Fallzahlrückgang robotischer Eingriffe im Vergleich zu 2019 bei 70% der Kliniken vor allem im zweiten Jahresquartal 2020 (64,7%) zur Folge. Dies wurde auf Personalmangel nichtchirurgischer Disziplinen (Anästhesie 35,3%, OP-Pflege 35,3%, Intensivmedizin 17,6%), interne Regularien (58,8%) und begrenzte Intensiv- oder Überwachungskapazitäten (47,1%) zurückgeführt. Die COVID-19-Pandemie führte in der robotischen Ausbildung teilweise bei der Assistenz am OP-Tisch (23,5%) und der Assistenz an der zweiten Konsole (42,9%) zu einem kompletten Ausbildungsstopp. Ausschlaggebend für diese Entwicklung war überwiegend der Rückgang der Operationszahlen.

**Schlussfolgerung:**

Die Robotik wird mittlerweile in einem breiten Spektrum der Viszeralchirurgie an Kliniken mit unterschiedlichen Versorgungsschwerpunkten in Deutschland eingesetzt. Der relative Anteil der Eingriffe am Gesamtspektrum ist allerdings noch gering. Roboterassistierte Eingriffe sind expertenfokussiert und es bestehen sehr heterogene Ausbildungskonzepte. Ein Lernerfolg mit konstanten und niedrigen Konversionsraten ist nach wenigen Jahren mit zunehmender Erfahrung zu erkennen. Die COVID-19-Pandemie hatte insgesamt einen negativen Einfluss auf die robotischen OP-Fallzahlen und die damit verbundenen Ausbildungsmöglichkeiten bei freien chirurgischen Personalressourcen. Hier ist eine kreative Gestaltung optimierter Ausbildungsmodalitäten erforderlich.

## Abstract (en)

**Background:**

Robotic procedures are gaining more and more importance in visceral surgery and seem to develop into an indispensable tool in minimally invasive visceral surgery. In 2020 the COVID-19 pandemic caused unexpected changes in daily surgical routines with still ongoing challenges. We evaluated the impact of the COVID-19 pandemic on robotic visceral procedures and the associated training provided in Germany.

**Material and methods:**

We performed a thorough evaluation of German hospitals and identified 89 surgical departments performing robotic visceral procedures. After extensive topic-related literature search an online questionnaire was developed. It included 35 questions referring to all relevant topics on robotic surgery, such as training programs and influence of the COVID-19 pandemic. The survey was sent via email three times to each department. Descriptive and subgroup analysis were performed.

**Results:**

We reported a response to our questionnaire from 22 (24.7%) surgical departments and17 questionnaires were analyzable. The vast majority of them weresurgical departments of university hospitals (58.8%), 17.6% maximum care clinics and 23.5% main care clinics. Robotic procedures were performed for the upper gastrointestinal tract (UGI 88.2%), the hepatopancreaticobiliary system (HPB 82.4%), in the colorectal region (94.1%) and for hernias (35.3%). The relative proportion of robotic operations in comparison to all visceral procedures was between 0.3% and 15.4%. The average conversion rate was 4.6 ± 3.2% referring to 2020. All participating clinics used the robotic DaVinci® system (Intuitive Surgical Inc., CA, USA). In summary 22 robotic systems were used mainly in an interdisciplinary setting (82.4%). For teaching purposes, 7 departments (41.2%) provided a second robotic console. On average 13.2 ± 6.5% of surgeons per clinic were involved in robotic procedures. Defined operating room (OR) teams (82.4%) consisted of consultants, specialists and residents. Team training for surgeons and OR nurses was mainly (52.9%) based on clinic-specific programs. Due to the COVID-19 pandemic the number of robotic procedures decreased in 70.0% of the participating departments compared to 2019 with the highest decline reported during the second quarter of 2020 (64.7%). Referring to this, staff shortage of non-surgical disciplines (anesthesiologists 35.3%, OR nurses 35.3%, intensive care medics 17.6%), COVID-19-specific regulations (58.8%) and limited capacities of intensive and intermediate care (47.1%) were specified as underlying causes. Due to the COVID-19 pandemic, caused by a decline in numbers of robotic procedures, robotic training was paused completely in assistance at the operating table in 23.5% and at the second console in 42.9%.

**Conclusion:**

Robotic visceral surgery is already implemented with a broad spectrum of operations in many German clinics of different care levels; however, the relative proportion of robotic procedures is low, when compared to the overall caseload of each clinic. Training concepts are heterogeneous and focused on experts. In surgeons with growing experience in robotic surgery, conversion rates are recorded to be very low. There was a negative impact on robotic case numbers and training provided in 2020 caused by the COVID-19 pandemic. Therefore, a further endorsement of robotic training programs and an improvement of training designs seem to be essential tools in order to enforce robotic procedures in visceral surgery.

## Hintergrund

Am 11.03.2020 wurde die weltweite Ausbreitung von COVID‑19 zur Pandemie erklärt [1]. In Deutschland wurde am 31.01.2020 die Meldepflicht nach § 6 und § 7 des Infektionsschutzgesetzes eingeführt. Die COVID-19-Pandemie hatte spätestens ab diesem Zeitpunkt den klinischen und chirurgischen Alltag wesentlich verändert. Mit dem 16.03.2020 traten umfangreiche Maßnahmen zur deutschlandweiten Eindämmung der COVID-19-Pandemie mit Reduktion des öffentlichen und sozialen Lebens in Kraft [2].

Der Einsatz von Operationsrobotern in der Viszeralchirurgie erfährt in Deutschland einen stetigen Zuwachs [3]. Er ist hier ein integraler Bestandteil der digitalen Innovation in der Chirurgie [4]. Allerdings verändert der Einsatz komplexer Technologie im Operationssaal das chirurgische Vorgehen sowohl im Hinblick auf das operative Vorgehen, als auch die Interaktion der Mitarbeiter. Dies bedarf spezifischer Trainingskonzepte, um den Einsatz der Roboter sicher und effizient zu gestalten [5, 6].

Die COVID-19-Pandemie hat persistierend Auswirkungen auf den chirurgischen Alltag, weshalb wissenschaftliche Evaluationen dieser besonderen Situation notwendig sind, um den resultierenden Herausforderungen qualifiziert und adäquat begegnen zu können [7]. Die hier präsentierte Studie basiert auf einer Statuserhebung zum Einsatz der Robotik in der Viszeralchirurgie in Deutschland im Jahr 2020. Hierbei wurden die Profile der einbezogenen Kliniken, die verwendeten Robotersysteme, das Eingriffsspektrum, der Einsatzes von Personal, sowie die damit verbundenen robotischen Ausbildungskonzepte beleuchtet. Der Einfluss der COVID-19-Pandemie auf diese relevanten Aspekte der roboterassistierten Viszeralchirurgie wurde evaluiert und hieraus resultierende kritische Entwicklungen dargestellt.

## Material und Methoden

### Literaturrecherche

Via PubMed erfolgte eine umfangreiche Literaturrecherche zu den folgenden Stichworten und deren Syntax: „coronavirus“/„COVID 19 pandemic“, „general surgery“, „robotic“, „residency training“, „teaching“, „Germany“. Des Weiteren wurden Daten folgender Homepages verwendet: Robert-Koch-Institut (RKI) Homepage [8] und Dashboard [9], John Hopkins University Dashboard [10], Bundesregierung Deutschland [11], Ourworldindata [12]. Auf Basis dieser Datenlage, insbesondere den Informationen für das Jahr 2020 mit den zu diesem Zeitpunkt aktuellen Empfehlungen zum Umgang mit der COVID-19-Pandemie im chirurgischen Bereich [13, 14], wurden die Forschungsfragen unter Inanspruchnahme der fakultätsinternen Expertise zum Infektionsgeschehen seitens des Instituts für Medizinische Mikrobiologie und Krankenhaushygiene formuliert. Für das stationäre COVID-19-Patientenaufkommen (Normal- und Überwachungsstation) wurden vier Stufen vorgegeben: niedrig (< 50 Fälle im Jahr 2020), moderat (50–199 Fälle im Jahr 2020), hoch (200–800 Fälle im Jahr 2020) und sehr hoch (> 800 Fälle im Jahr 2020).

### Digitaler Fragebogen

Der verwendete Fragebogen enthielt neben allgemeingültigen Informationen zu den teilnehmenden Kliniken drei Inhaltsbereiche (Robotik in Deutschland, COVID-19-Pandemie, robotische Ausbildung) mit insgesamt 35 Fragen. Die Gütekriterien der Konstruktvalidität, der Durchführungs- und Auswertungsobjektivität sowie die Reliabilität des Fragebogens mit Ausnahme der Interraterreliabilität aufgrund der einmaligen Teilnahme jeder Klinik waren durch die standardisierte Gestaltung des Fragebogens und der Umfragebedingungen via E‑Mail gewährleistet. Da die Daten über die Plattform google forms mit eigenem E‑Mail-Account (viszeralchir.uk.magdeburg@gmail.com) erhoben werden sollten, wurden Pretests und Modifikationen der Fragebogen- und Antwortensets inklusive der Fragebogeneinleitung durchgeführt. Unter den 35 Fragen entsprach Frage Nummer 5 (Vorhandensein eines OP-Roboters) der internen Kontrolle im Sinne eines Ausschlusskriteriums bei negativer Antwort. Die kalkulierte Bearbeitungszeit für den gesamten Fragebogen betrug 10–15 Min. Eine Anonymisierung der Umfrageteilnehmer war gewährleistet.

### Klinikrecherche und Umfrage

Die Teilnehmerrekrutierung erfolgte über eine webbasierte Recherche zu Kliniken mit Homepageangaben zu einem Operationsroboter im Bereich der Viszeralchirurgie. Inklusive der Universitätsklinika wurden 89 Kliniken in Deutschland identifiziert, welche ein Robotersystem für die Viszeralchirurgie im Jahr 2020 einsetzten. Insgesamt wurden drei E‑Mails in unterschiedlichen Zeitintervallen mit der Bitte um Teilnahme an der Studie versandt (initial: 09.02.2021, Reminder 08.03.2021, 2. Reminder 09.06.2021). Die Umfrage wurde zum 30.06.2021 geschlossen.

### Auswertung und Statistik

Nach Extraktion der Daten aus google forms über Microsoft Excel 2021 und Transformation in IBM® SPSS Statistics Version 26 wurde eine deskriptiv statistische Analyse sowie Subgruppenanalyse nach der jeweiligen Versorgungsstufe – Universitätsklinikum (UK), Maximal- (MV) oder Schwerpunktversorger (SV) – durchgeführt. Des Weiteren erfolgte eine gesonderte Betrachtung der Kliniken entsprechend des Anschaffungsjahres des OP-Roboters: Anschaffung des OP-Roboters im Kalenderjahr 2020 (Gruppe 1), Anschaffung des OP-Roboters vor dem Kalenderjahr 2020 (2015–2019; Gruppe 2). Hintergrund dieser Subgruppenanalyse war die Tatsache, dass die Kliniken der Gruppe 1 unter COVID-19-Pandemie-Bedingungen mit der robotischen Viszeralchirurgie begonnen hatten. Zu den roboterassistierten hepatopankreatikobiliären (HPB) Eingriffen wurden Leber- und Pankreasoperationen zusammengefasst. Die onkologischen und funktionellen Eingriffe am oberen Gastrointestinaltrakt (OGIT) inklusive der Adipositaschirurgie, welche bei der Datenauswertung dieser Kategorie zugeordnet wurde, wurden zusammengefasst sowie Kolon- und Rektumeingriffe als kolorektaler Bereich (KRK) dargestellt. Durch die Fragebogenkonstellation resultierte, dass drei Kliniken ohne feste OP-Teams keine Fragen zu Rotationen der OP-Teams beantworten konnten. Die intraoperative Assistenz am OP-Tisch wird im weiteren als Tischassistenz bezeichnet. Aufgrund der nicht ausreichenden Anzahl der auswertbaren Fragebögen erfolgte eine deskriptive statistische Auswertung ohne Signifikanzanalysen.

## Ergebnisse

Der höchste Rücklauf an ausgefüllten Fragebögen wurde nach der initialen Teilnahmeanfrage (09.02.2021) per E‑Mail (9/89; 10,1 %) verzeichnet, gefolgt von der Rückmeldung von sechs weiteren Kliniken (6/89; 6,7 %) nach der zweiten Erinnerung. Drei Kliniken gaben Probleme bei der Öffnung des Fragebogens an und zwei Universitätskliniken hatten zum Zeitpunkt der Umfrage keinen Roboter. Somit hat inklusive der eigenen Institution knapp ein Viertel der angeschriebenen Kliniken (22/89; 24,7 %) auf die Studienanfrage reagiert und es konnten die Daten von 17 Kliniken (19,1 %) ausgewertet werden. Die im Folgenden präsentierten Ergebnisse beziehen sich daher auf 17 Kliniken, die über einen OP-Roboter verfügten, an der Umfrage teilgenommen haben und einen auswertbaren Fragebogen zurückgesandt hatten.

### Bestandsaufnahme der robotischen Viszeralchirurgie in Deutschland 2020

Die wesentlichen Charakteristika der an der Umfrage teilgenommenen Kliniken in Abhängigkeit der Versorgungsstufe sind in Tab. 1 zusammengefasst. Zwei der angegebenen Werte der durchgeführten viszeralchirurgischen Operationen im Gesamtkollektiv im Jahr 2020 waren aufgrund der sehr niedrigen absoluten Zahlen nicht plausibel (*n* = 4 und *n* = 5). Diese wurden daher für diese Variablenauswertung ausgeschlossen. Die Beteiligung der ärztlichen Mitarbeiter bei robotischen Eingriffen lag im Mittel bei 13,2 ± 6,5 % (UK: 12,2 ± 7,9 %; MV: 11,6 ± 0,4 %; SV: 16,9 ± 4,1 %).

**Table 1:** 

Parameter	UK *(n* *=* *10)*	MV *(n* *=* *3)*	SV *(n* *=* *4)*	Gesamt *(n* *=* *17)*
	*n* (%) oder M ± SD	*n* (%) oder M ± SD	*n* (%) oder M ± SD	*n* (%) oder M ± SD
Umfrageteilnehmer	10 (58,8)	3 (17,6)	4 (23,5)	17 (100,0)
**Personal**				
*Gesamte Mitarbeiteranzahl Ärzte*	47 ± 16	26 ± 1	23 ± 10	38 ± 17
OÄ	12 ± 4	8 ± 1	6 ± 1	10 ± 4
FÄ	12 ± 5	7 ± 0	7 ± 2	10 ± 5
AÄ	23 ± 9	11 ± 1	10 ± 8	18 ± 10
*An Robotik beteiligte Ärzte*	5 ± 2	3 ± 0	4 ± 1	4 ± 2
**Robotische Technik**				
*Anschaffungsjahr OP-Roboter*				
Gruppe 1: 2020	2 (11,8)	3/17 (17,6)	2 (11,8)	7 (41,2)
Gruppe 2: 2015–2019	8 (47,1)	–	2 (11,8)	10 (58,8)
– 2019	2 (11,8)	–	–	2 (11,8)
– 2018	–	–	1 (5,9)	1 (5,9)
– 2017	1 (5,9)	–	–	1 (5,9)
– 2016	3 (17,6)	–	–	3 (17,6)
– 2015	2 (11,8)	–	1 (5,9)	3 (17,6)
*Durchschnittliche Nutzung des OP-Roboters in Jahren*	3,7 ± 2,0	1,0 ± 0	2,8 ± 2,4	3,0 ± 2,1 (2,0)
*Interdisziplinäre Nutzung*	7 (70,0)	3 (100,0)	4 (100,0)	14 (82,4)
*Zwei OP-Roboter pro Klinik*^a^	4 (80,0)	–	1 (20,0)	5 (29,4)
*2. Konsole*^b^	6 (85,7)	–	1 (14,3)	7 (41,2)
**Operationen**				
*Gesamte Anzahl viszeralchirurgischer Operationen*^c^	2690 ± 1598	3033 ± 58	2125 ± 943	2608 ± 1256
*Gesamte Anzahl robotischer Operationen*	121 ± 99	62 ± 3	25 ± 18	88 ± 85,7
*Prozentualer Anteil robotischer Operationen*	7,0 ± 5,2	2,0 ± 0,1	1,5 ± 1,2	4,6 ± 4,6
Gruppe 1	2,8 ± 0	2,0 ± 0,1	1,2 ± 1,3	1,9 ± 0,9
Gruppe 2	7,6 ± 5,3	–	1,8 ± 1,4	6,3 ± 5,3
*Konversionsrate in Prozent*				
2020	5,4 ± 3,1	3,0 ± 0	4,0 ± 4,5	4,6 ± 3,2
2019	5,0 ± 2,3	–	2,5 ± 3,5	4,5 ± 2,6
*Organsysteme*				
OGIT	9 (90,0)	3 (100,0)	3 (75,0)	15 (88,2)
– Onkologisch	9 (90,0)	3 (100,0)	2 (50,0)	14 (82,4)
– Funktionell	5 (50,0)	3 (100,0)	3 (75,0)	11 (64,7)
HPB	9 (90,0)	3 (100,0)	2 (50,0)	14 (82,4)
– Leber	7 (70,0)	–	1 (25,0)	8 (47,1)
– Pankreas	9 (90,0)	3 (100,0)	1 (25,0)	13 (76,5)
KRK	9 (90,0)	3 (100,0)	4 (100,0)	16 (94,1)
– Kolon	7 (70,0)	3 (100,0)	3 (75,0)	13 (76,5)
– Rektum	9 (90,0)	3 (100,0)	4 (100,0)	16 (94,1)
Hernien	3 (30,0)	–	3 (75,0)	6 (35,3)

*UK* Universitätsklinik, *MV* Maximalversorger, *SV* Schwerpunktversorger, *OÄ* Oberärzt*innen, *FÄ* Fachärzt*innen, *AÄ* Assistenzärzt*innen, *OGIT* oberer Gastrointestinaltrakt, *HPB* hepatopankreatikobiliäres System* KRK* kolorektales System

^a^Grundgesamtheit (Anzahl Kliniken): *n* = 5, ^b^Grundgesamtheit (Anzahl Kliniken): *n* = 7, ^c^Grundgesamtheit (Anzahl Kliniken): *n* = 15

In Deutschland wurde im Jahr 2020 bezugnehmend auf die teilnehmenden Kliniken vorwiegend das DaVinci®-System für roboterassistierte viszeralchirurgische Eingriffe verwendet. Insgesamt waren 22 OP-Roboter in den 17 Kliniken installiert, d. h. in fünf Kliniken wurden zwei Roboter benutzt. Hinsichtlich der DaVinci®-Modelle wurden folgende Generationen eingesetzt: Model Xi in 59,1 % (13/22; UK: *n* = 10; SV: *n* = 3), Model X in 27,3 % (6/22; UK: *n* = 2; MV: *n* = 3; SV: *n* = 1) und Model Si in 13,6 % (3/22; UK: *n* = 2; SV: *n* = 1). In den fünf Kliniken mit zwei verfügbaren OP-Robotern kamen unterschiedliche Generationen der DaVinci®-Serie parallel zum Einsatz: zweimal die Kombination aus den Generationen „X und Xi“ und einmal die Kombination aus „Si und Xi“. In zwei Kliniken war der DaVinci® Xi doppelt verfügbar.

In der Subgruppenanalyse Gruppe 1 vs. Gruppe 2 bezüglich der robotischen Konversionsraten zeigten sich keine wesentlichen Unterschiede für das Jahr 2020 (4,0 ± 3,3 vs. 5,1 ± 3,2; UK: 4,0 ± 4,2 vs. 5,8 ± 3,0; SV: 5,5 ± 6,4 vs. 2,5 ± 3,5).

**Figure 1:**
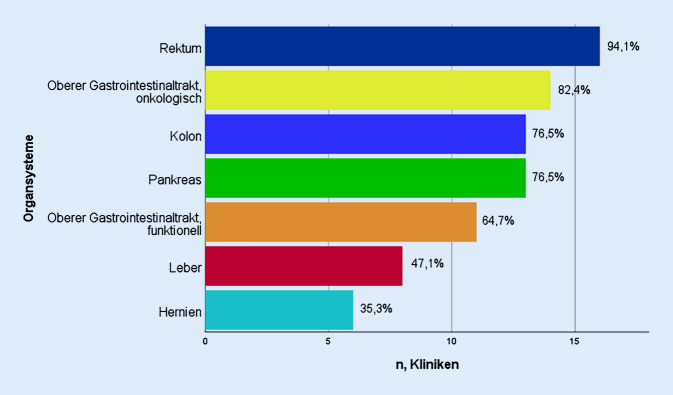

Das roboterassistierte Eingriffsspektrum umfasste ein weites Spektrum der Viszeralchirurgie vom oberen Gastrointestinaltrakt über Leber‑, Pankreas- bis zu kolorektalen Operationen und der Hernienversorgung (Abb. 1). Dabei wurden Eingriffe am KRK am häufigsten (94,1 %), gefolgt von Operationen am OGIT (88,2 %) und etwas weniger am HPB (82,4 %) durchgeführt (Tab. 1). Am wenigsten wurde der Roboter für die Chirurgie an der Leber (47,1 %) und der Hernienversorgung (35,3 %) eingesetzt. Kliniken aller Versorgungsstufen setzten den Roboter für Operationen am OGIT, HPB oder KRK ein. Unterschiede waren allerdings in der Anwendung der Organsysteme entsprechend den Versorgungsstufen erkennbar. So wurde der Roboter bei den SV zu 50 % bei onkologischen Eingriffen am OGIT oder zu 25 % bei Eingriffen an Leber oder Pankreas verwendet, während dies bei den UK zu 90 % und 70 % der Fall war (Tab. 1). Der prozentuale Anteil robotischer Operationen an allen viszeralchirurgisch durchgeführten Eingriffen lag minimal bei 0,3 % und maximal bei 15,4 %.

Alle sieben Kliniken, die im Jahr 2020 die robotische Viszeralchirurgie begonnen hatten, operierten am kolorektalen Bereich und OGIT mit dem Roboter. Von den acht Kliniken, die Lebereingriffe roboterassistiert durchführten, hatten sieben den OP-Roboter vor dem Jahr 2020 (Gruppe 2) angeschafft. Zusammengefasst führten fünf der 17 Kliniken (29,4 %) über 100 viszeralchirurgische Eingriffe robotisch im Jahr 2020 durch. Dabei lag die Konversionsrate bei 80,0 % (8/10) der Kliniken mit robotischer Erfahrung von bis zu sechs Jahren (Gruppe 2) unter 5 % für die Jahre 2019 und 2020.

### Bestandsaufnahme der Ausbildung in der robotischen Viszeralchirurgie 2020

Die Aspekte der viszeralchirurgischen Ausbildung mit Schwerpunkt Robotik der an der Umfrage teilgenommenen Kliniken in Abhängigkeit der Versorgungsstufe sind in Tab. 2 dargestellt. Für die Ergebnisanalyse zur zweiten Konsole in Tab. 2 besteht die Grundgesamtheit aus 7 Kliniken (UK: *n* = 6; SV: *n* = 1).

**Table 2:** 

Parameter	UK *(n* *=* *10)*	MV *(n* *=* *3)*	SV *(n* *=* *4)*	Gesamt *(n* *=* *17)*
	*n* (%)	*n* (%)	*n* (%)	*n* (%)
**Strukturiertes Ausbildungskonzept vorhanden**	10 (100,0)	3 (100,0)	4 (100,0)	17 (100,0)
Ärzte	1 (10,0)	1 (33,3)	1 (25,0)	3 (17,6)
OP-Pflege	1 (10,0)	–	–	1 (5,9)
Ärzte + OP-Pflege	8 (80,0)	2 (66,7)	3 (75,0)	13 (76,5)
**Schulungen**				
Fachgesellschaften	1 (10,0)	–	–	1 (5,9)
Klinikintern	3 (30,0)	–	–	3 (17,6)
Firmenbezogen	1 (10,0)	–	2 (50,0)	3 (17,6)
Klinikintern + firmenbezogen	4 (40,0)	3 (100,0)	2 (50,0)	9 (52,9)
Alle Optionen	1 (10,0)	–	–	1 (5,9)
**Feste OP-Teams**	8 (80,0)	2 (66,7)	4 (100,0)	14 (82,4)
**OP-Tisch**				
*Assistenz*				
Alle Ärztegruppen	4 (40,0)	1 (33,3)	1 (25,0)	6 (35,3)
OÄ	5 (50,0)	1 (33,3)	3 (75,0)	9 (52,9)
FÄ	5 (50,0)	1 (33,3)	2 (50,0)	8 (47,1)
AÄ	8 (80,0)	1 (33,3)	3 (75,0)	12 (70,6)
OP-Pflege	5 (50,0)	3 (100,0)	1 (25,0)	9 (52,9)
*Rotationen*				
Intervall < 12 Monate	3 (30,0)	2 (66,7)	–	5 (29,4)
Intervall > 12 Monate	2 (20,0)	–	–	2 (11,8)
Individuelle Intervalle	3 (30,0)	–	3 (75,0)	6 (35,3)
Keine Rotation	–	–	1 (25,0)	1 (5,9)
**2. Konsole**^a^				
*Assistenz*				
Alle Ärztegruppen	1 (16,7)	–	–	1 (14,3)
OÄ	4 (66,7)	–	1 (14,3)	5 (71,4)
FÄ	3 (50,0)	–	–	3 (42,9)
AÄ	1 (16,7)	–	–	1 (14,3)
*Rotationen*				
Intervall < 12 Monate	2 (33,3)	–	–	2 (28,6)
Intervall > 12 Monate	2 (33,3)	–	–	2 (28,6)
Individuelle Intervalle	1 (16,7)	–	1 (100,0)	2 (28,6)

*UK* Universitätsklinik, *MV* Maximalversorger, *SV* Schwerpunktversorger, *OÄ* Oberärzt*innen, *FÄ* Fachärzt*innen, *AÄ* Assistenzärzt*innen

^a^Grundgesamtheit (Anzahl Kliniken): *n* = 7

Insgesamt gaben 76,5 % der Kliniken an, über ein strukturiertes Ausbildungskonzept für Ärzte und Pflegeteams zu verfügen. Dabei wurden in 52,9 % klinikinterne und firmenbezogene Schulungen durchgeführt. Schulungen durch Fachgesellschaften wurden nur in 5,9 % der Fälle genutzt, während rein firmenbezogene oder klinikinterne Fortbildungen in 17,6 % in die Ausbildung einbezogen wurden. Feste Teams für die roboterassistierten Operationen waren in 82,4 % der Kliniken vorhanden. Der Personaleinsatz am robotischen OP-Tisch und der zweiten Konsole wurde überwiegend aus einer Kombination der unterschiedlichen Ärztegruppen und der OP-Pflege realisiert. Am häufigsten waren Assistenzärzt*innen (70,6 %, 12 Kliniken) als Tischassistenz involviert. Lediglich in drei der zwölf Kliniken (17,6 %) waren sie die einzige als Tischassistenz eingesetzte Personalgruppe. In zwei Kliniken (11,8 %) assistierten nur Oberärzt*innen am OP-Tisch. In drei Kliniken (17,6 %) wurde die Tischassistenz ausschließlich durch das OP-Pflegepersonal übernommen, wobei die OP-Pflege insgesamt in 52,9 % der Kliniken als Tischassistenz eingesetzt wurde. In 5,9 % der Kliniken war hinsichtlich der Tischassistenz keine Rotationsmöglichkeit vorgesehen. Die Assistenz an der zweiten Konsole war im Wesentlichen den Oberärzt*innen (71,4 %) vorbehalten. Fachärzt*innen (42,9 %) und Assistenzärzt*innen (14,3 %) wurden hier deutlich weniger eingesetzt. Allerdings boten alle Kliniken mit einer zweiten Konsole Rotationen in regelmäßigen oder individuellen Abständen an.

### Nationale Auswirkungen der COVID-19-Pandemie auf die Patientenversorgung in der (robotischen) Viszeralchirurgie 2020

Für die Betrachtung der Änderung der OP-Fallzahlen wurde das Jahr 2020 zugrunde gelegt und auf das Jahr 2019 bezogen. In Tab. 3 werden die wesentlichen Aspekte zur Auswirkung der COVID-19-Pandemie auf die Patientenversorgung der an der Umfrage teilgenommenen Kliniken in Abhängigkeit der Versorgungsstufe dargestellt. Ein niedriges stationäres COVID-19-Patientenaufkommen trat in keiner der teilnehmenden Kliniken auf. In 47,1 % der Kliniken fand sich ein moderates (50–199 Fälle) oder hohes (200–800 Fälle) Patientengut an COVID-19-infizierten Patienten*innen. Etwas weniger als die Hälfte der Kliniken (47,1 %) verzeichnete während dieser Zeit keinen Personalmangel. Wenn ein solcher auftrat, betraf dies vor allem die Anästhesie (35,3 %) oder OP-Pflege (35,3 %). Personalmangel bei Chirurg*innen trat am seltensten auf (11,8 %). Bei detaillierter Betrachtung des Personalmangels waren in sechs Kliniken (6/17; 35,3 %) mehrere Berufsgruppen betroffen. Während der COVID-19-Pandemie führte die Mehrzahl der Kliniken (58,8 %) lediglich Notfalleingriffe durch. Ein elektives Operationsprogramm fand nur in 11,8 % der Kliniken statt. Das zweite Quartal des Jahres 2020 wurde am häufigsten bezüglich einer Änderung der OP-Fallzahlen (11/17; 64,7 %) genannt. Intraoperative Modifikationen bei robotischen Operationen zum Schutz des Personals führten 35,3 % der Kliniken während der COVID-19-Pandemie ein. Am häufigsten fand dabei eine kontrollierte Gasevakuation (17,6 %) als isolierte Maßnahme Anwendung. Das intraoperative Personal wurde nur bei 11,8 % reduziert. Eine Mitarbeitersurveillance der gesamten Viszeralchirurgie fand in 52,9 % der Kliniken während der COVID-19-Pandemie statt.

**Table 3:** 

Parameter	UK *(n* *=* *10)*	MV *(n* *=* *3)*	SV *(n* *=* *4)*	Gesamt *(n* *=* *17)*
	*n* (%)	*n* (%)	*n* (%)	*n* (%)
**Stationäres COVID-19-Aufkommen in 2020**				
50–199 Fälle (moderat)	5 (50,0)	–	3 (75,0)	8 (47,1)
200–800 Fälle (hoch)	4 (40,0)	3 (100,0)	1 (25,0)	8 (47,1)
> 800 Fälle (sehr hoch)	1 (10,0)	–	–	1 (5,9)
**Personalmangel**				
Kein Personalmangel	4 (40,0)	3 (100,0)	1 (25,0)	8 (47,1)
Chirurgie	1 (10,0)	–	1 (25,0)	2 (11,8)
Anästhesie	3 (30,0)	–	3 (75,0)	6 (35,3)
OP-Pflege	3 (30,0)	–	3 (75,0)	6 (35,3)
Intensivmedizin	–	–	3 (75,0)	3 (17,6)
**Operationsdringlichkeit viszeralchirurgischer Eingriffe**				
Notfall	6 (60,0)	2 (66,7)	2 (50,0)	10 (58,8)
Dringlich elektiv	–	1 (33,3)	1 (25,0)	2 (11,8)
Elektiv und im Notfall	2 (20,0)	–	–	2 (11,8)
**Intraoperative Modifikationen bei robotischen Eingriffen**				
Mehrere Maßnahmen gleichzeitig	4 (40,0)	–	2 (50,0)	6 (35,3)
Kontrollierte Gasevakuation	3 (30,0)	–	–	3 (17,6)
Reduktion des intraoperativen Personals	2 (20,0)	–	–	2 (11,8)
Keine Maßnahmen	2 (20,0)	2 (66,7)	2 (50,0)	6 (35,3)
**Mitarbeitersurveillance**				
Freiwillig	3 (30,0)	–	2 (50,0)	5 (29,4)
Nur für Robotik	–	–	1 (25,0)	1 (5,9)
Für gesamte Viszeralchirurgie	6 (60,0)	1 (33,3)	2 (50,0)	9 (52,9)
**OP-Fallzahländerung**				
*Gesamt viszeralchirurgisch 2020/2019*^a^				
Zunahme um 10–20 %	1 (10,0)	–	–	1 (5,9)
Keine	2 (20,0)	–	–	2 (11,8)
Abnahme < 10 %	1 (10,0)	2 (20,0)	1 (25,0)	4 (23,5)
Abnahme um 10–20 %	3 (30,0)	1 (10,0)	3 (75,0)	7 (41,2)
Abnahme um 21–30 %	3 (30,0)	–	–	3 (17,6)
*Robotisch 2020/2019 (Gruppe 2*^*b*^*)*				
Zunahme um 10–20 %	1 (12,5)	–	–	1 (10,0)
Keine	2 (25,0)	–	–	2 (20,0)
Abnahme < 10 %	1 (12,5)	–	–	1 (10,0)
Abnahme um 10–20 %	1 (12,5)	–	–	1 (10,0)
Abnahme um 21–30 %	2 (25,0)	–	1 (50,0)	3 (30,0)
Abnahme um 31–40 %	1 (12,5)	–	1 (50,0)	2 (20,0)
*Robotik: von OP-Fallzahländerung betroffene Organsysteme (Gruppe 2*^b^*)*				
OGIT	4 (50,0)	–	–	4 (40,0)
– Onkologisch	3 (37,5)	–	–	3 (30,0)
– Funktionell	1 (12,5)	–	–	1 (10,0)
HPB	4 (50,0)	–	–	4 (40,0)
– Leber	2 (25,0)	–	–	2 (20,0)
– Pankreas	3 (37,5)	–	–	3 (30,0)
KRK	4 (50,0)	–	1 (50,0)	5 (50,0)
– Kolon	3 (37,5)	–	–	3 (30,0)
– Rektum	3 (37,5)	–	1 (50,0)	3 (30,0)
Hernien	1 (12,5)	–	1 (50,0)	2 (20,0)

*UK* Universitätsklinik, *MV* Maximalversorger, *SV* Schwerpunktversorger, *OGIT* oberer Gastrointestinaltrakt, *HPB* hepatopankreatikobiliäres System, *KRK* kolorektales System

^a^Grundgesamtheit: alle Kliniken (*n* = 17), ^b^Grundgesamtheit: Kliniken der Gruppe 2 (Anschaffungsjahre OP-Roboter 2015–2019; *n* = 10)

**Figure 2:**
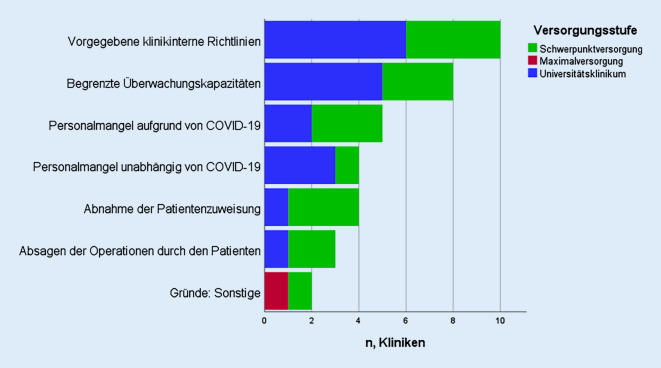

Von vierzehn Kliniken (82,3 %) wurden Rückgänge der viszeralchirurgischen OP-Fallzahlen von bis zu 30 % im Vergleich zum Jahr 2019 angegeben. Für die Robotik wurde in 70 % der Kliniken ein Fallzahlrückgang der Operationen bis zu 40 % angegeben. Betroffen waren hiervon alle Organsysteme gleichermaßen (OGIT, HPB, KRK, Hernien), welche unter Anwendung des Roboters operiert wurden. In Abb. 2 sind die Gründe für die robotische OP-Fallzahländerung in Abhängigkeit der Versorgungsstufe zusammengefasst. Klinikinterne Richtlinien und die begrenzte Anzahl von Überwachungskapazitäten waren dabei die führenden angegebenen Ursachen. Bei zehn Kliniken (58,8 %) wurden mehrere Aspekte für die veränderte OP-Fallzahlen robotischer Eingriffe zugrunde gelegt, während in den übrigen sieben Kliniken nur einzelne Aspekte zum Tragen kamen.

### Nationale Auswirkungen der COVID-19-Pandemie auf die Ausbildung in der (robotischen) Viszeralchirurgie 2020

Bei 47,1 % der an der Studie teilgenommenen Kliniken hatte die COVID-19-Pandemie einen Einfluss auf die viszeralchirurgische Ausbildung. Bei den SV waren hiervon sogar 100 % betroffen. Die Ausbildung am Roboter wurde dadurch bei 41,2 % der Kliniken an der Tischassistenz und bei 14,3 % an der Ausbildung an der zweiten Konsole nicht beeinflusst. Allerdings gab es bei 23,5 % der Kliniken bei der Tischassistenz und bei 42,9 % bei der Ausbildung an der zweiten Konsole einen kompletten Ausbildungsstopp (Tab. 4). Als wesentlicher Grund für diesen Ausbildungsstopp wurde ein Rückgang der Gesamtfallzahl an elektiven viszeralchirurgischen Operationen sowie Infektionsschutz des Personals vor allem bei der Ausbildung an der zweiten Konsole genannt.

**Table 4:** 

	UK *(n* *=* *10)*	MV *(n* *=* *3)*	SV *(n* *=* *4)*	Gesamt *(n* *=* *17)*
	*n* (%)	*n* (%)	*n* (%)	*n* (%)
Auswirkungen: Ja	4 (40,0)	–	4 (100,0)	8 (47,1)
Auswirkungen: Nein	6 (60,0)	3 (100,0)	–	9 (52,9)
**Robotische Ausbildung**				
*OP-Tisch*				
Keine Auswirkungen	4 (40,0)	3 (100,0)	–	7 (41,2)
Negative Auswirkungen				
– Kompletter Ausbildungsstopp	2 (20,0)	–	2 (50,0)	4 (23,5)
– Aufgrund reduzierter OP-Fallzahlen	4 (40,0)	–	3 (75,0)	7 (41,2)
– Aufgrund reduzierten Personals	2 (20,0)	–	1 (25,0)	3 (17,6)
*2. Konsole*				
Keine Auswirkungen	1 (16,7)	–	–	1 (14,3)
Negative Auswirkungen				
– Kompletter Ausbildungsstopp	2 (33,3)	–	1 (100,0)	3 (42,9)
– Aufgrund reduzierter OP-Fallzahlen	2 (33,3)	–	–	2 (28,6)

*UK* Universitätsklinik, *MV* Maximalversorger, *SV* Schwerpunktversorger

## Diskussion

Nur 24,7 % der angeschriebenen Kliniken reagierten auf die Studienanfrage. Es waren 19 % der rückgemeldeten Fragebögen auswertbar. Dieses Ergebnis ließ sich leider durch eine dreimalige Anfrage nicht relevant optimieren. Daher sind die vorliegenden Ergebnisse nicht repräsentativ, sodass die Aussagekraft und Interpretationsmöglichkeiten limitiert sind. Allerdings konnten dennoch über drei Versorgungsstufen hinweg Kliniken für die Studie gewonnen werden, was eine Einschätzung der aktuellen Situation in der Anwendung der robotischen Systeme in der Viszeralchirurgie und der gegenwärtigen Ausbildungskonzepte erlaubt. Der zeitliche Aufwand für die Beantwortung der Umfrage war eher gering. Daher stellt sich die Frage nach dem Grund für die limitierte Teilnahme der angefragten Institutionen. Insbesondere Universitätsklinika sollten Ressourcen für derartige Studien vorhalten können. Möglicherweise sind Onlineumfragen in Deutschland noch nicht hinreichend etabliert und als wissenschaftliches Instrument akzeptiert. Auch in anderen Bereichen zeigt sich, dass auf nationalem Niveau der Wissensaustausch anhand niedriger Teilnahmequoten bei deutschen Umfragen deutlich eingeschränkt ist [15]. Für europäische Länder wie Belgien und Frankreich waren ebenfalls niedrige Antwortraten unter Mitarbeitern des Gesundheitswesens nachweisbar [16]. Das Problem niedriger Rücklaufquoten bei Onlineumfragen variiert international sehr stark, wobei Deutschland teilweise nicht explizit im Ländervergleich erwähnt wird [16]. Von der Verwendung digitaler Optionen zu wissenschaftlichem Arbeiten kann die klinische Forschung zeitgemäß profitieren. Eine flächendeckende Akzeptanz kann eine notwendige Transparenz in Deutschland und die damit verbundenen Entwicklungsmöglichkeiten gewährleisten. International können dadurch sowohl wissenschaftliche Sichtbarkeit, als auch Kooperationsmöglichkeiten gefördert werden.

Viszeralchirurgische robotische Operationen wurden in den teilnehmenden Kliniken lediglich durch Modelle der Firma Intuitive® (Santa Clara, CA, USA) durchgeführt. Es existieren mittlerweile aber auch erste Erfahrungsberichte in Deutschland mit anderen Anbietern von OP-Robotern [17, 18], beispielsweise dem Senhance® Robotic System [19]. Zudem wurde deutlich, dass bei überwiegend interdisziplinärer Nutzung gleichzeitig mehrere Generationen der DaVinci®-Modelle im Einsatz sind, für die jeweils eigene operative Zugangswege im Sinne der Trokarplatzierungen, Andocken des Roboters und intraoperative Vorgehensweisen notwendig sind. Für jede Generation von OP-Robotern und Herstellern ist eine Standardisierung perioperativer Abläufe für die alltägliche Praxis erforderlich. Dies ist nicht nur im Rahmen von Ausbildungen mit einem hohen Aufwand verbunden. Da das Angebot von OP-Robotersystemen inter-/national steigt [20], kann die gegenwärtige klinische Situation der robotischen Viszeralchirurgie als eine ökonomische und wissenschaftliche Übergangsphase betrachtet werden. Es ist zu erwarten, dass die Dynamik der technischen Entwicklung die Umsetzung im klinischen Alltag aufgrund der Zunahme der Anbieter übersteigt. Diese Situation verstärkt die Problematik der strukturellen Implementierung der Robotik im chirurgischen Alltag im Rahmen der Facharztausbildung. Strukturierte, herstellerunabhängige Ausbildungskonzepte sind daher zu fordern, welche basierend auf unserer Umfrage noch unterrepräsentiert sind. Unter COVID-19-Bedingungen kam es dennoch zu einem Start des Roboterprogramms in sieben Kliniken (41,2 %) im Jahr 2020. Ein Investitions- bzw. Weiterentwicklungsstopp lag demzufolge nicht vor, obwohl rückläufige Patientenzahlen für elektive Operationen zu verzeichnen waren. Es ist anzunehmen, dass die Planungen für diese Investitionen bereits lange im Vorfeld der Pandemie getätigt und deswegen umgesetzt wurden.

Unsere Studie hat gezeigt, dass mit der zunehmenden Erfahrung verbunden mit geschultem Personal robotische Operationen erfolgreich etabliert werden können. Die Konversionsraten lagen konstant durchschnittlich bei unter 5 %, ohne dass die COVID-19-Pandemie einen relevanten Einfluss im Sinne einer Steigerung hatte. Selbst Kliniken, die erst 2020 mit robotischen Eingriffen starteten (Gruppe 1), gaben durchschnittlich keine höheren Konversionsraten an. Eine Standardisierung der innerklinischen Abläufe mit enger Indikationsstellung und spezifischer Patientenselektion garantiert in der Initialphase die Überwindung der notwendigen Lernkurve und gewährt die Patientensicherheit. Dies ermöglicht es, im Verlauf komplexere Eingriffe auf Basis der erworbenen Erfahrung durchzuführen. Besonders bei Eingriffen am hepatobiliären System helfen dabei spezifische Modifikationen der chirurgischen Techniken [21]. In diesem Zusammenhang ist ergänzend zu erwähnen, dass über die letzten Jahre der Anteil robotischer Eingriffe der einzelnen Kliniken am Gesamtkollektiv der viszeralchirurgischen Operationen (4,6 ± 4,6 %) sehr niedrig war. Diese Beobachtung fand sich selbst in der Subgruppe der Kliniken, die bereits vor 2020 den OP-Roboter einsetzten (6,3 ± 5,3 %). Demzufolge ist hier ein hohes Potenzial zur Steigerung des Einsatzes der OP-Roboter in der Viszeralchirurgie erkennbar. Interessant wäre in diesem Zusammenhang der Anteil laparoskopischer Eingriffe im Vergleich zu den robotischen Operationen im zeitlichen Verlauf, um zu verstehen, ob ein Wechsel von der Laparoskopie hin zur Robotik stattgefunden hat.

Hinsichtlich der Tischassistenz bei robotischen Operationen war die Personaleinteilung sehr heterogen, wobei in ca. 71 % Assistenzärzt*innen involviert waren. Die Ausbildung an der zweiten Konsole ist überwiegend an Fach- und Oberärzt*innen gebunden. Dies zeigt, dass besonders die operative Ausbildung an der Konsole immer noch „Experten“ vorbehalten ist. Der Nachwuchs muss aber künftig besser in diese Ausbildungskonzepte mit einbezogen werden. Der bisherige Expertenbezug spiegelt sich auch bei Rotationen wider, da diese an der zweiten Konsole wesentlich seltener und hauptsächlich individuell erfolgten. Am OP-Tisch waren Rotationen zwar häufiger als an der zweiten Konsole, allerdings ist auch hier kein Muster unter den teilnehmenden Kliniken ableitbar.

Der Konfiguration der intraoperativen interprofessionellen OP-Teams wurde nach Angaben der teilnehmenden Kliniken durch interdisziplinäre Schulungen unter Einbeziehung der OP-Pflege und der Chirurg*innen Rechnung getragen. Immerhin waren bei 76,5 % die Pflegekräfte in Ausbildungskonzepte mit einbezogen und absolvierten in über 50 % auch die Tischassistenz. Beim Ausbildungskonzept der Kliniken spielten Kursangebote von Fachgesellschaften eine untergeordnete Rolle. Da die Firmen bei den teilnehmenden Kliniken einheitliche, die Kliniken selbst intern unterschiedliche Vorgehensweisen hatten, ist das Schulungsvorgehen für roboterassistierte Eingriffe folglich als sehr heterogen einzustufen.

Die COVID-19-Pandemie hatte einen relevanten negativen Einfluss auf die robotische Ausbildung. Aufgrund der heterogenen Ausbildungskonzepte mit Schwerpunkt auf klinikinternen Schulungen waren qualitativ alle involvierten Personen von Assistenz- bis Oberärzt*innen betroffen. Die reduzierte OP-Fallzahl aggravierte diese Problematik, sodass es teilweise sogar zu einem kompletten Ausbildungsstopp kam. Dies zeigt, dass nicht nur strukturierte Ausbildungskonzepte für roboterassistierte Operationen in der Viszeralchirurgie etabliert werden müssen, sondern das auch nach modernen, alternativen Methoden zur Ausbildungsgestaltung fern vom Patienten gesucht werden muss.

Im direkten Vergleich der Kliniken, die bereits vor dem Jahr 2020 roboterassistiert operiert hatten, zeigte sich eine insgesamt ähnliche Fallzahländerung wie bei allen anderen Kliniken mit stärkerer negativer Auswirkung auf die robotischen Eingriffszahlen. Der Rückgang an Eingriffen war im Besonderen klinikinternen Regularien geschuldet, welche aufgrund des klinischen COVID-19-Aufkommens in den Kliniken notwendig waren. Eine Ursache ist dabei im Ausfall von Personal in der Anästhesie und im Pflegebereich zu suchen. Aber auch reduzierte postoperative Überwachungsmöglichkeiten waren hier maßgeblich. Deutschlandweit konnte ein Defizit von 7 % bei stationären onkologischen Patienten und eine Zunahme von 21 % bei der ambulanten Betreuung onkologischer Patienten verursacht durch die COVID-19-Pandemie verzeichnet werden [22]. Es wird angenommen, dass es pandemisch bedingt zu verzögerten bzw. aufgeschobenen Diagnostikmöglichkeiten mit entsprechenden Folgen für therapeutische Maßnahmen kam und zudem, dass Patienten eine stationäre Behandlung aufgrund der COVID-19-Situation vermieden bzw. dieser zurückhaltend gegenüberstanden. Dies reflektiert die angespannte Situation im onkologischen Bereich. Die COVID-19-Pandemie betraf weiterhin auch die kooperierenden Fachdisziplinen wie die Endoskopie, denen ebenfalls nur begrenzte Kapazitäten zur Verfügung standen, was sich gleichermaßen auf die Fallzahlen operativer onkologischer Eingriffe auswirkte. Vor dem Hintergrund pandemischer Herausforderungen konnte jedoch lokal beispielhaft demonstriert werden, dass durch Umstrukturierungen unter gewissen Voraussetzungen, wie Bereitstellung ausreichender Schutzausrüstung, eine Aufrechterhaltung der Funktionalität möglich ist [23]. Um das Personal zu schützen, wurden bei 35,3 % der Kliniken spezifische Maßnahmen (z. B. kontrollierte Gasevakuation) bei robotischen Eingriffen während der COVID-19-Pandemie eingeführt. Eine weitere perioperative Modifikation der teilnehmenden Kliniken an der Studie bestand in einer Mitarbeitersurveillance bezüglich einer COVID-19-Infektion. In Kombination mit einem Patientenscreening ist damit eine hohe chirurgische Qualität bei minimalem Infektionsrisiko gewährleistet [24]. Bezugnehmend auf das kalkulierbare Infektionsrisiko und den Vorteilen minimal-invasiver Eingriffe [25] könnte die roboterassistierte Chirurgie gerade für den Einsatz in Krisenzeiten zur Gewährleistung der Qualität komplexer onkologischer Eingriffe unter Berücksichtigung der Patienten- und Mitarbeitersicherheit sowie der intraoperativen Arbeitsbelastung von Vorteil sein [26–28].

In der Summe ist festzuhalten, dass die Robotik mittlerweile in einem breiten Spektrum der Viszeralchirurgie eingesetzt wird. Der relative Anteil der Eingriffe am Gesamtspektrum ist allerdings noch gering. Roboterassistierte Eingriffe sind expertenfokussiert und es bestehen sehr heterogene Ausbildungskonzepte. Die COVID-19-Pandemie hatte insgesamt einen negativen Einfluss auf die robotischen OP-Fallzahlen und die Ausbildungsmöglichkeiten bei freien chirurgischen Personalressourcen. Hier ist eine Restrukturierung mit Gestaltung optimierter Ausbildungsmodalitäten erforderlich.

## Anhang

### Fragebogen

*Titel:*

Anonyme Umfrage: Einfluss der COVID-19-Pandemie auf die roboterassistierte Viszeralchirurgie 2020

*Einleitung:*

Die COVID-19-Pandemie hat unseren chirurgischen Alltag komplett verändert. Ob dies Einfluss auf innovative operative Verfahren hat, wollen wir mit der folgenden Umfrage klären. Sie hat zum Ziel, den Einfluss der COVID-19-Pandemie auf die roboterassistierte Viszeralchirurgie und die damit verbundene Ausbildung zu analysieren.

Der zeitliche Rahmen für die Beantwortung der Fragen umfasst ca. 10–15 Min.

*Legende Antwortmöglichkeiten*:

- Multiple-Choice (MC): nur eine Auswahlmöglichkeit
- Kurzantworttext (F)
- Mehrfachauswahl (A): mehrere Auswahlmöglichkeiten, max. der Anzahl der Antworten entsprechend

*Fragen:*

Frage 1: Welche Versorgungsstufe leistet Ihre Institution? Antwort: MC: Grund- und Regelversorgung, Schwerpunktversorgung, Maximalversorgung, Universitätsklinikum
Frage 2: Wie viele Assistenzärzt*innen arbeiten in Ihrer Abteilung/Klinik? Antwort: F
Frage 3: Wie viele Fachärzt*innen arbeiten in Ihrer Abteilung/Klinik? Antwort: F
Frage 4: Wie viele Oberärzt*innen arbeiten in Ihrer Abteilung/Klinik? Antwort: F
Frage 5: Werden bei Ihnen viszeralchirurgische Operationen unter Anwendung eines OP-Roboters durchgeführt? Antwort: MC: Ja, Nein → falls Nein: Umfrageende
Frage 6: Wie viele Chirurgen*innen (als Operateur, als Assistenz) führen roboterassistierte Eingriffe durch? Antwort: F
Frage 7: Wann wurde bei Ihnen die erste robotisch assistierte viszeralchirurgische Operation durchgeführt? [Datum] Antwort: F
Frage 8: Wie viele OP-Roboter stehen aktuell für die Viszeralchirurgie zur Anwendung zur Verfügung? Antwort: MC: 1, 2, 3, > 3
Frage 9: Wird der OP-Roboter interdisziplinär genutzt? Antwort: MC: Ja, Nein
Frage 10: Mit welchem Roboter arbeiten Sie in Ihrer Klinik? (Mehrfachauswahl möglich) Antwort: A: Da Vinci Si® von Intuitive, Da Vinci X® von Intuitive, Da Vinci Xi® von Intuitive, Da Vinci SP® von Intuitive, Weitere
Frage 11: Steht Ihnen eine 2. Konsole (zu Ausbildungszwecken) zur Verfügung? Antwort: MC: Ja, Nein
Frage 12: Für welche Organe führen Sie roboterassistierte Eingriffe durch? (Mehrfachauswahl möglich) Antwort: A: Oberer Gastrointestinaltrakt (onkologisch), Oberer Gastrointestinaltrakt (funktionell), Leber, Pankreas, Kolon, Rektum, Hernien, Weitere
Frage 13: Wie viele viszeralchirurgische Operationen haben Sie im Jahr 2020 durchgeführt? Antwort: F
Frage 14: Wie viele roboterassistierte Operationen haben Sie im Jahr 2020 durchgeführt? Antwort: F
Frage 15: Wie hat sich die Gesamt-OP-Fallzahl 2020 im Vergleich zum Jahr 2019 in der Viszeralchirurgie verändert? Antwort: MC: kein Rückgang, Abnahme < 10 %, Abnahme 10–20 %, Abnahme 21–30 %, Abnahme 31–40 %, Abnahme 41–50 %, Abnahme > 50 %, Zunahme < 10 %, Zunahme 10–20 %, Zunahme 21–30 %, Zunahme > 30 %
Frage 16: In welchem Quartal war die OP-Fallzahländerung 2020 im Vergleich zum Jahr 2019 am höchsten? Antwort: MC: Erstes Quartal 2020 (Januar–März), Zweites Quartal 2020 (April–Juni), Drittes Quartal 2020 (Juli–September), Viertes Quartal 2020 (Oktober–Dezember)
Frage 17: Wie hat sich die robotische OP-Fallzahl 2020 im Vergleich zum Jahr 2019 in der Viszeralchirurgie verändert? Antwort: MC: kein Rückgang, Abnahme < 10 %, Abnahme 10–20 %, Abnahme 21–30 %, Abnahme 31–40 %, Abnahme 41–50 %, Abnahme > 50 %, Zunahme < 10 %, Zunahme 10–20 %, Zunahme 21–30 %, Zunahme > 30 %
Frage 18: Welches Organsystem war vorrangig von der robotischen Fallzahländerung 2020 betroffen? (Mehrfachauswahl möglich) Antwort: A: Oberer Gastrointestinaltrakt (onkologisch), Oberer Gastrointestinaltrakt (funktionell), Leber, Pankreas, Kolon, Rektum, Hernien, Weitere
Frage 19: Bitte nennen Sie den vorrangigen Grund für die Veränderung der robotischen OP-Fallzahl im Jahr 2020. (Mehrfachauswahl möglich) Antwort: A: Keine Fallzahländerung, Personalmangel unabhängig von der COVID-19-Pandemie, Personalmangel aufgrund von Krankheit inkl. Corona-Quarantänefällen, Vorgegebene Richtlinien im Rahmen der COVID-19-Pandemie (innerklinische Regulationen, Aufnahmestopp etc.), Begrenzte Überwachungskapazitäten (IMC, Intensivstation), Absagen der Operationen durch den Patienten, Abnahme der Patientenzuweisung
Frage 20: Falls Personalmangel der Grund für die robotische Fallzahländerung 2020 war, welche Berufsgruppe/n waren vorrangig betroffen? (Mehrfachauswahl möglich) Antwort: A: Kein Personalmangel, Chirurgie, Anästhesie, OP-Pflege, Intensivmedizin
Frage 21: Wie viele COVID-19-Fälle wurden in Ihrem Krankenhaus 2020 stationär behandelt? (Normalstation + Überwachungsstation) Antwort: MC: < 50 Fälle, 50–199 Fälle, 200–800 Fälle, > 800 Fälle
Frage 22: Von den chirurgisch versorgten Patienten, unter welchen Umständen haben Sie COVID-19-positive Patienten operiert? Antwort: MC: Es wurden keine COVID-19-Fälle operiert., Nur im Notfall, (Dringend) Elektiv, Elektiv und im Notfall
Frage 23: Welche perioperativen Aspekte haben Sie im Rahmen der COVID-19-Pandemie für roboterassistierte Operationen modifiziert? (Mehrfachauswahl möglich) Antwort: A: Keine Modifikationen, Aktive Reduktion der intraoperativ anwesenden Personenanzahl, Kontrollierte Gasevakuation zur Reduktion der Aerosolbildung und -ausbreitung, Reduzierter intraabdominaler Druck (z. B. 8 mm Hg oder niedriger), Minimierte verwendete Stromstärke, Vermeiden von Strom‑/Ultraschallapplikation intraoperativ, Für die gesamte (Viszeral‑) Chirurgie: engmaschige Mitarbeitersurveillance zur Aufrechterhaltung der Betriebsfähigkeit, Nur für roboterassistierte Eingriffe: engmaschige Mitarbeitersurveillance zur Aufrechterhaltung der Betriebsfähigkeit, Freiwillige (!) Teilnahme am Mitarbeiterscreening/-surveillanceprogramm (z. B. regelmäßige Abstriche)
Frage 24: Wie hoch war die Konversionsrate für roboterassistierte Eingriffe in der Viszeralchirurgie für das Jahr 2019? [%] Antwort: F
Frage 25: Wie hoch war die Konversionsrate für roboterassistierte Eingriffe in der Viszeralchirurgie für das Jahr 2020? [%] Antwort: F
Frage 26: Haben Sie ein strukturiertes Ausbildungsprogramm für die roboterassistierte Viszeralchirurgie in Ihrer Abteilung/Klinik? Antwort: MC: Ja – für Ärzte*innen, Ja – für das OP-Pflegepersonal, Ja – für Ärzte*innen und das OP-Pflegepersonal, Nein
Frage 27: Wie ist Ihr Ausbildungsprogramm für roboterassistierte Viszeralchirurgie konzipiert? (Mehrfachauswahl möglich) Antwort: A: Durch Fachgesellschaften, Klinikintern, Firmenbezogen, Weitere
Frage 28: Wer assistiert in Ihrer Abteilung/Klinik am OP-Tisch bei robotischen Eingriffen? (Mehrfachauswahl möglich) Antwort: A: Oberärzt*innen, Fachärzt*innen, Assistenzärzt*innen, OP-Pflegepersonal
Frage 29: Wer assistiert in Ihrer Abteilung/Klinik an der 2. Konsole bei robotischen Eingriffen? (Mehrfachauswahl möglich) Antwort: A: Keine 2. Konsole vorhanden, Oberärzt*innen, Fachärzt*innen, Assistenzärzt*innen, OP-Pflegepersonal
Frage 30: Sind bei Ihnen feste OP-Teams für die roboterassistierten Eingriffe eingeteilt? Antwort: MC: Ja, Nein
Frage 31: Rotieren die OP-Teams für die Assistenz am OP-Tisch in festen zeitlichen Abständen? Antwort: MC: Ja – Rotationszeitraum < 12 Monate, Ja – Rotationszeitraum > 12 Monate, Nein – generell keine Rotation, Nein – nur individuelle Rotation in unterschiedlichen Zeitabständen
Frage 32: Rotieren die OP-Teams für die Assistenz an der 2. Konsole in festen zeitlichen Abständen? Antwort: MC: Ja – Rotationszeitraum < 12 Monate, Ja – Rotationszeitraum > 12 Monate, Nein – generell keine Rotation, Nein – nur individuelle Rotation in unterschiedlichen Zeitabständen
Frage 33: Hatte die COVID-19-Pandemie 2020 einen Einfluss auf die chirurgischen Ausbildungsmöglichkeiten am OP-Roboter? Antwort: MC: Ja, Nein
Frage 34: Wie hat sich die Ausbildung am OP-Tisch bei roboterassistierten Operationen 2020 verändert? (Mehrfachauswahl möglich) Antwort: A: Keine Veränderung – Die Ausbildung konnte wie zuvor fortgeführt werden., Negativ – Die Ausbildung wurde komplett pausiert., Negativ – Aufgrund niedrigerer OP-Fallzahlen war die Ausbildung nicht in gleichem Ausmaß möglich., Negativ – Aufgrund von Personalmangel war die Ausbildung nicht in gleichem Ausmaß möglich., Positiv – Die Ausbildung konnte sogar verbessert bzw. ausgebaut werden., Weitere
Frage 35: Wie hat sich die Ausbildung an der 2. Konsole bei roboterassistierten Operationen 2020 verändert? (Mehrfachauswahl möglich) Antwort: A: Keine 2. Konsole vorhanden., Keine Veränderung – Die Ausbildung konnte wie zuvor fortgeführt werden., Negativ – Aufgrund niedrigerer OP-Fallzahlen war die Ausbildung nicht in gleichem Ausmaß möglich., Negativ – Aufgrund von Personalmangel war die Ausbildung nicht in gleichem Ausmaß möglich., Positiv – Die Ausbildung konnte sogar verbessert bzw. ausgebaut werden., Weitere

*Zu Unterthemen zugeordnete Fragen:*

- Klinikprofile mit Schwerpunkt Robotik in Deutschland: Fragen 1–4, 6–9, 10, 12–14, 24–25
- Ausbildung in der robotischen Viszeralchirurgie: Fragen 11, 26–35
- Auswirkungen der COVID-19-Pandemie: Fragen 15–23
